# The Book World of Medicine and Science

**Published:** 1897-11-13

**Authors:** 


### THE BOOK WORLD OF MEDICINE AND SCIENCE.

Heart Disease: With Special Reference to Prognosis and Treatment. By Sir William H. Broadbent, Bart., M.D.Lond., F.R.S., F.R.C.P., and John F. H. Broadbent, M.A., M.D. (Oxon.), M.R.C.P. (London: Baillière, Tindall, and Cox. 1897. Price 10s. 6d.)

This book is based on certain lectures delivered by Sir William Broadbent in 1884 and in 1891 dealing more especially with prognosis. These have been rearranged by Dr. John Broadbent, who has also engrafted upon them the subject of treatment, so that as the work now stands it may be taken as a complete and reliable manual dealing with heart disease from the standpoint which is of most interest to the practising physician, namely, that of treatment and prognosis. It is thus essentially a modern book, although it contains the teachings of one who has long been known in connection with the subject of which it treats. The book commences with a chapter on the clinical examination of the heart, after which valvular diseases, their etiology, symptoms, prognosis, and treatment are successively dealt with.

In discussing the different symptoms met with in heart disease, that most important one, dropsy, receives a due share of careful consideration. The causes of dropsical effusion, as ascertained by experiment, are stated to be obstruction to the return of venous blood, loss of tone in the vessels, induced by destruction of the nerves of the part, and a wasting condition of the blood, and it is maintained that the condition which is most certainly and conspicuously present in valvular disease is obstruction to the return of venous blood to the heart. The difficulties in the way of accepting this view, as set forth by Walshe, are plainly stated, and an attempt is made to controvert them, or at least to explain them away—not, however, as we think, with great success. Probably it will be found that dropsy, like many other things, is the consequence of a summation of causes.

The chapters on prognosis and on the general treatment of heart disease are admirable, and give many useful hints, especially in regard to digitalis, its uses, its abuses, and its substitutes. A careful description of the signs, the prognosis, and the treatment of the various individual valvular lesions is given; congenital and other affections are dealt with, and then we come to what really is in many cases the key to both treatment and prognosis, namely, structural disease of the heart, taking the form of hypertrophy, dilatation, and fatty degeneration. These are treated of with considerable fulness. It is worthy of note that throughout the book the authors group together dilatation and hypertrophy as "compensatory changes," and that in regard to conditions of regurgitation it is definitely stated that dilatation is a necessary part of compensation. All this is good and true, but we think they might usefully have gone further, and drawn a much sharper line than they have done between the dilatation which is necessary in order that a proper quantity of blood shall be projected forwards with each systole, and that destructive dilatation which is associated with a systole. That there is such a distinction to be drawn every clinician knows, and that the authors recognise it is clear enough, and under these circumstances it seems a pity that, speaking of dilatation in general, they should lay it down that "even more important than the anatomical condition is the physiological or functional condition, the special characteristic of which is that the ventricle does not complete its systole, but only expels a portion of its contents." This is true in many cases—in fact, it is the very essence of heart failure—but it can hardly be held to be true of that form of dilatation which, combined with a proportionate hypertrophy, compensates for a regurgitation. If, however, we weed out cases of compensatory dilatation, and take this chapter as applying only to dilatation which is either independent of valvular disease or is in excess of what is necessary for compensation, it is an admirable description of the manner in which hearts give way. The book, in fact, is full of suggestiveness, and is one which ought to be widely read by medical practitioners. A careful study of its pages ought greatly to lessen the frequency with which that wretched term "weak heart" is palmed off upon patients as explanatory of their symptoms—"a vague term," as the authors say, "which covers the entire ground from temporary debility to disease inevitably and imminently fatal."

Economics, Anæsthetics, and Antiseptics in the Practice of Midwifery. By Haydn Brown, L.R.C.P., L.R.C.S., Ed. (London: J. and A. Churchill, 7, Great Marlborough Street. 1897. 103 pages. Price 2s. 6d.)

"The object of these pages is to go carefully over the simple fundamental management of a midwifery case, and to give principles and methods of procedure according as our more advanced knowledge of everything suggests methods and means, so that the perfected superstructure as set forth in so many standard works may have a good bottom to stand upon." We quote this sentence in full, not only because it supplies us with the only reason we can find for the publication of this book, but as an example of the simple and lucid style of the writer. The book contains, in the first place, an elaborate treatise on deportment. It discusses at great length the paramount importance in the lying-in chamber of "tact, method, and style." A variety of other questions are discussed—*e.g.*, the feelings of the "naturally shy and sensitively proper woman," septicæmia, anæsthetics, use of instruments, nurses, midwives, and antiseptics. We learn nothing, however, of any great novelty, and we could have "made shift to get along" in our professional career without this book.

Notes on the Special Hygiene (Physical and Mental) of Childhood and Youth. By Thomas More Madden, M.D., F.R.C.S.E., M.A.O., Royal University of Ireland, Obstetric Physician, and Gynæcologist Mater Miserecordia Hospital; Consulting Physician to the Hospital for Children, Dublin; Consultant and ex-Master National Lying-In Hospital. (Fannin and Co., Dublin. Baillière, Tindall, and Cox, London. 1897. 68 pages.)

This paper has as its object the summarising of "certain general principles applicable in the correction of certain popular errors prevalent" on points of hygiene in connection with childhood and youth. The question of the general management and feeding of infants is first treated. The author advises that "tinned milks should not be recommended unless good fresh milk, procured from cows the immunity of which from tuberculosis has been proved by Koch's tuberculin test, is not obtainable." This seems to us a counsel of perfection. The section on moral and mental culture in early childhood is excellent. With reference to the habit among children of sucking or smoking tobacco the author draws his illustration from Portugal. The secrecy with which the habit is indulged by English children would seem to point to a healthy public opinion on this point among English parents. In the section on the training of girls, the author's estimate of the physical and mental instability of girls and young women seems to be in accordance with that usually held by the gynæcologist. The plea for more physical exercise should be sown broadcast amongst the authorities in our modern girls' schools, though we venture to suspect that the very infrequency in these institutions of those neuroses with which the author threatens their pupils for their sins is a reason for their being all too deaf to this and similar cries of "wolf."

